# Elective Inguinal Hernia Surgery in the Setting of Unfollowed Congenital Complete Heart Block With No Postponement

**DOI:** 10.7759/cureus.10865

**Published:** 2020-10-09

**Authors:** Kyle S Varkoly, Roxana Beladi, Steven Ritrosky

**Affiliations:** 1 Cardiology, Kansas City University of Medicine and Biosciences, Joplin, USA; 2 General Surgery, Kansas City University of Medicine and Biosciences, Joplin, USA; 3 Anesthesiology, US Anesthesia Partners, Fort Myers, USA

**Keywords:** congenital complete heart block, preoperative assessment and risk management, temporary pacemaker, metabolic equivalents, case report

## Abstract

Most preoperatively discovered complete heart block cases without cardiac clearance in a non-emergent situation are managed with deferral of elective surgery until a cardiology workup can be completed. The medical consequences of surgical delays can manifest in increased costs to the healthcare system via the treatment of more advanced disease, often requiring more intense and more costly treatment in addition to the emotional burden of delay on a patient that has been waiting months for a particular surgery. Delays in surgery have real impacts on patient health outcomes, hospital finances, and patient satisfaction.

We present a rare case in which a proactive anesthesiologist was able to take measures to stratify patient safety risk and safely prevent the delay of the surgery in an asymptomatic and unfollowed congenital third-degree heart block patient. The anesthesiologist demonstrates the use of established guidelines for non-elective noncardiac surgery to safely and effectively prevent the delay of an elective inguinal hernia repair in the setting of a situation that normally warrants its delay.

Using these pre- and intraoperative measures, the anesthesiologist was able to prevent the delay of elective surgery, and this should set a precedent of the necessary steps involved to safely manage a patient with an unfollowed third-degree congenital heart block.

## Introduction

Congenital atrioventricular heart block is a rare and heterogeneous disorder with an estimated incidence of one for every 22,000 live births [1]. A subset of these patients, including the one presented, is asymptomatic until adulthood, at which time he/she may become symptomatic or asystolic upon exposure to anesthetic agents.

The 2014 European Society of Cardiology (ESC)/European Society of Anesthesiology (ESA) guidelines on non-cardiac surgery state that the preoperative establishment of temporary or permanent cardiac pacing may be appropriate for asymptomatic adults with complete heart block [2]. Additionally, the use of metabolic equivalents (MET) in the preoperative diagnostic workup for patients undergoing noncardiac surgery provides another tool for the anesthesiologist to use in this situation [3]. Through the accurate use of these guidelines, surgical delay was prevented, and the patient was able to be treated successfully for his inguinal hernia with no complications.

## Case presentation

The patient is an obese, but otherwise healthy, 34-year-old male presenting for a robotic laparoscopic inguinal hernia repair. A preoperative electrocardiogram (EKG) revealed a third-degree atrioventricular (AV) block, which was not recorded in the preceding surgical preoperative note. Our patient knew of his condition but did not disclose this information to anyone until it was discovered via history, physical, and chart review on the day of surgery. He was not aware of the risks associated with the anesthetic procedure and did not have an established cardiologist following his condition. The last echocardiogram was nine years ago in 2011, which revealed no systolic, diastolic, or valvular abnormalities. His last visit to a cardiologist was five years ago in 2015, during which he obtained an EKG unchanged from the normal preoperative EKG he received on the day of his elective surgery. While his EKG on the day of surgery was normal, anesthesiology preoperative history and physical revealed that he has a history of congenital complete AV block. He has no current pacemaker in place and his complete AV block was not followed by a cardiologist when he presented for surgery.

Our patient's past medical history was significant for bradycardia, chest pain, dyspnea on exertion, hypertension, and complete heart block. However, even though our patient's past medical history was significant for the above, his present medical status and vitals were within normal limits, with the exception of his elevated blood pressure at 169/110. History and physical revealed that he was able to do heavy work around the house, such as lifting furniture and running for exercise, which would classify the patient as having greater than 10 METs. However, even though he was presently asymptomatic with greater than 10 METs, the fact that he had no current establishment of cardiac care warranted further investigation, especially with his concurrent hypertension. A cardiologist was called preoperatively for a consult.

The patient was thoroughly counseled on his condition and anesthetic risk. Following a consult with the attending cardiologist, his general surgeon, and the attending anesthesiologist, the decision was affirmed to continue with the elective surgery with the aid of a perioperative temporary venous pacer and central line placement. In most circumstances, cases like this would be deferred, but with the use of a temporary venous pacer, a MET of greater than 10, and a cardiology consult, it was avoided.

**Figure 1:**
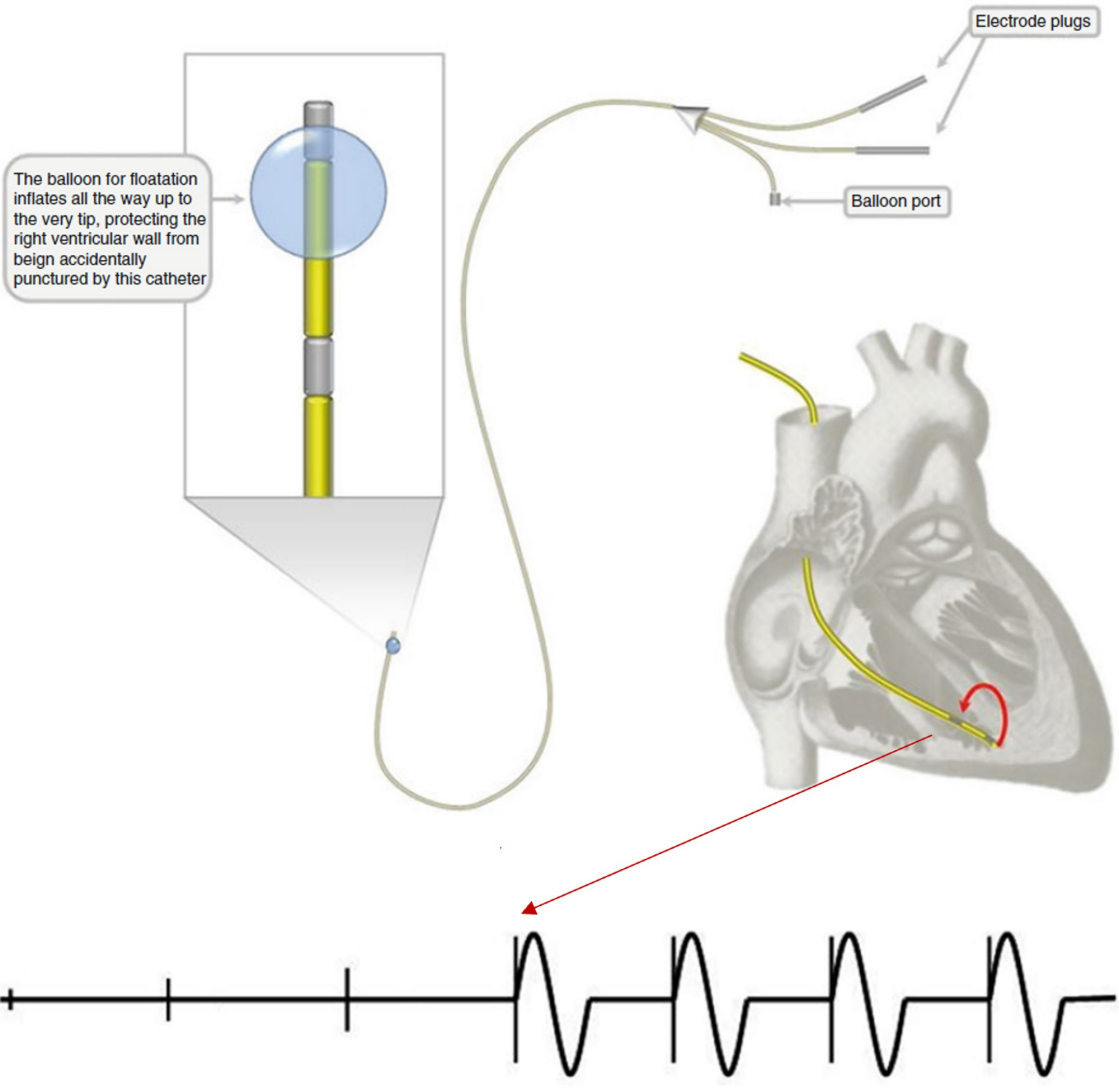
Capture of RV electrophysiology when the transvenous catheter contacts the RV endocardium The figure was used with the written permission of Springer Nature and altered by author Kyle Varkoly for the educational purposes of this paper. RV: right ventricular

A 6F introducer sheath was inserted into central access via the subclavian vein for transvenous pacing. A 5F balloon-tipped temporary venous pacer catheter (covered in a sterile sleeve) was inserted 20 centimeters into the sheath and then floated under direct fluoroscopy guidance when in the hybrid room. A biopatch was placed at the entry site and an introducer sutured in place. The 5F balloon-tipped pacer catheter with balloon inflated (covered in a sterile sleeve) was advanced using fluoroscopic guidance until it reached the right ventricle apex at approximately 40 centimeters. The balloon was deflated and the capture of pacer function was verified down to 1.5 milliamps as illustrated in Figure 1 [4-5]. The pacer controller was then placed at 10 milliamps output for intraoperative pacing.

**Figure 2:**
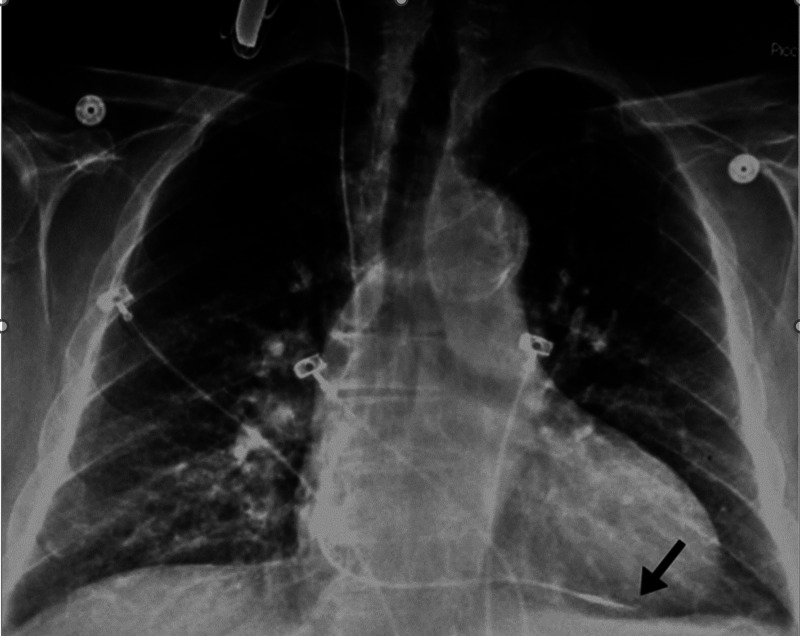
CXR confirming the placement of the transvenous pacemaker tip in the right ventricle (arrow) The figure was used with the written permission of publisher Springer Nature. Permissions were needed, as this CXR was published by Springer Nature. CXR: chest X-ray

The pacer catheter was then secured to the introducer sheath with a suture tie and stabilized to the shoulder. At the end of the procedure, the catheter remained anchored by sutures with a sterile dressing over the entry site. Chest X-ray (CXR) confirmed the placement of the transvenous pacemaker lead in the right ventricle, as demonstrated in Figure 2 [4-5]. The patient tolerated the procedure well and with no complications. Muscle relaxers used include intravenous (IV) succinylcholine. The anesthetics administered include IV propofol for induction with inhalational sevoflurane to aid in the maintenance of stage III anesthesia. The patient remained intraoperatively stable under general anesthesia, with a pacer wire throughout the surgery without complication. Postoperatively, the anesthesiologist removed the pacemaker and side port. The consulting cardiologist followed up outpatient with 24-hour telemetry monitoring.

## Discussion

In asymptomatic adults with adult congenital heart disease (ACHD), permanent pacing is recommended (Class IIa) per American College of Cardiology (ACC)/American Heart Association (AHA)/Heart Rhythm Society (HRS) guidelines [6]. Thus, it is reasonable to expect that such patients would already have a permanent pacer or at least need prior cardiac clearance before surgery day. Although our patient lacked both, strategic planning through the following three components: a temporary pacemaker, preoperative usage of METs, and a perioperative cardiac consult, physicians are better able to recognize when the continuation of surgery will yield a successful outcome. A previous case report with complete heart block uncovered on the day of surgery resulted in the abortion of the surgery intraoperatively due to the patient's bradycardia converting into a junctional escape rhythm and ultimately varying degrees of AV block. The author's discussions considered the deference of surgery until an electrophysiological study had been completed to locate the site of the complete heart block [7].

Our case depicts a unique approach of not immediately postponing surgery by using the three aforementioned key components. The use of METs via estimated via patient history has been proven by Weinstein et al. to be an underestimation of METs measured via exercise stress testing and thus serves as a more conservative assessment on the side of patient safety, lowering the threshold for further workup/deferring a procedure [8]. Using patient-estimated METs in our case allowed us to err on the side of caution when deciding to pursue the surgery. Our patient’s ability to achieve greater than or equal to 10 METs without symptoms was an excellent prognostic indicator per 2014 American College of Cardiology/American Heart Association (ACC/AHA) guidelines on perioperative cardiovascular evaluation and management of patients undergoing noncardiac surgery [3].

Another effective strategy is to call for an urgent cardiac consult when the patient's functional status is reassuring, to allow the possibility of continuing with the surgery. Immediately deciding to postpone a surgery can be unnecessary and cause real impacts on patient health outcomes, hospital finances, and resources [9]. In addition to the emotional and financial burden of the surgical delay to the patient, surgeons can be faced with backlogs of patients that can present with more severe cases in the end. In this case, the anesthesiologist would have to weigh the risk-benefit analysis in delaying the surgery for a beneficial outpatient cardiac consult versus the risk of the elective surgery turning non-elective if the inguinal hernia would turn incarcerated or strangulated, causing more harm to the patient in the process. Prompt cardiology consult and thorough action can save a patient and surgical team from having to reschedule, to the benefit of both the surgeon and patient involved.

Complete heart block, also known as third-degree AV block, is an issue due to the nonsequential depolarization of the atria and ventricles. The timing of pacemaker insertion for asymptomatic complete heart block remains a controversial topic, but all patients will eventually need at least a transvenous pacemaker [10]. Currently, it is a Class IIa recommendation for pacemaker insertion in a patient with complete heart block [6]. Pacing currently has a Class I recommendation only for patients with heart failure symptoms, a resting heart rate of less than 40 beats per minute when awake, or with ventricular pauses greater than three seconds [11]. Class I recommendations have not been presently made for patients with asymptomatic ACHD [3].

Bradycardia must be considered during volatile anesthesia administration in patients such as the one presented here with complete heart block. Volatile anesthetics, such as the sevoflurane used here, when combined with preexisting conduction abnormalities, can greatly enhance AV nodal blocking and lengthen the QT interval via the inhibition of voltage-gated sodium and L-type calcium channels [12]. A complete heart block has been reported during induction with propofol as the injectable anesthetic alone, particularly in elderly cases [13-14]. Care should also be taken when administering these commonly used anesthetics with commonly used muscle blockers, such as the succinylcholine used here, as a second-degree AV block has been reported to have converted to a third-degree in susceptible patients [15]. All aforementioned drugs require extensive monitoring during their administration in complete heart block patients.

## Conclusions

A previous case report called for the consideration of deference of surgery until an electrophysiological study has been done to locate the site of the complete heart block. Given the negative health, emotional, and socioeconomic causes of delaying elective and nonelective surgeries, this may not be the best option. While it may be needed in the specific case of newly uncovered heart block discovered intraoperatively, we can now see through the combined usage of the current European Society of Cardiology and the European Society of Anaesthesiology (ESC/ESA) guidelines of usage of a temporary pacemaker, combined with the established ACC/AHA guidelines for the preoperative usage of METs and perioperative cardiac consult, noncardiac elective surgery does not necessarily need to be postponed in the setting of unfollowed congenital complete heart block.
